# Temporal Reproducibility of a Genetic Algorithm–Derived Health Risk Score: Standardized Out-of-Fold Validation Framework (2021-2023)

**DOI:** 10.2196/85659

**Published:** 2026-04-21

**Authors:** Yoichiro Aoki, Hiroki Takeda, Kinichi Yokota, Ryoko Yoshida

**Affiliations:** 1 Yoshida Hospital-Keiyukai Medical Corporation, 1-2, Nishi 4-chome, 4-jyo, Asahikawa, Hokkaido, 070-0054, Japan, 81 166-23-0685; 2 Department of Cardiovascular Medicine, Yoshida Hospital- Keiyukai Medical Corporation, Asahikawa, Hokkaido, Japan; 3 Department of Gastroenterology, Yoshida Hospital-Keiyukai Medical Corporation, Asahikawa, Hokkaido, Japan

**Keywords:** genetic algorithm, health risk scoring, reproducibility, cross-validation, ROC, AUC, preventive medicine, area under the receiver operating characteristic curve

## Abstract

**Background:**

Genetic algorithm (GA)–based scoring has been proposed as a data-driven approach for health risk stratification . However, performance estimates may be inflated when preprocessing, optimization, and evaluation are not strictly separated within a prespecified validation framework. Demonstrating temporal reproducibility under a standardized out-of-fold (OOF) evaluation framework with transparent uncertainty quantification is therefore essential for ensuring translational reliability in preventive health screening.

**Objective:**

This study aimed to evaluate the temporal reproducibility of a GA-derived composite health risk score across three consecutive annual cohorts (2021‐2023) under a standardized OOF validation pipeline and to assess robustness to policy-driven structural HbA_1c_ missingness through a prespecified ON/OFF sensitivity analysis.

**Methods:**

Annual health examination datasets from 2021 (n=3744), 2022 (n=5153), and 2023 (n=5352) were analyzed using an identical preprocessing and modeling pipeline. Thirteen clinical indicators and eight lifestyle questionnaire variables were included as predictors. The outcome was based on an A–D grading framework and binarized using an OR rule across domains (grade ≥B in any domain). Continuous variables were median-imputed and standardized within each training fold to prevent information leakage. GA optimization was performed using fixed random seeds, and fitness estimation employed stratified K-fold cross-validation. Predicted probabilities were obtained by fitting logistic regression models to GA-derived composite scores within the OOF framework. Discrimination and overall predictive performance were quantified using the area under the receiver operating characteristic curve (AUC) and the Brier score calculated from OOF predicted probabilities. Uncertainty was estimated using 2,000-replicate percentile bootstrap resampling. A prespecified sensitivity analysis excluded HbA_1c_ while maintaining an identical evaluation framework.

**Results:**

OOF AUC values were stable across cohorts (2021: 0.810; 2022: 0.814; 2023: 0.812), with overlapping 95% percentile bootstrap confidence intervals. Brier scores ranged from 0.172 to 0.176. Exclusion of HbA_1c_ resulted in small changes in discrimination (median ΔAUC was ≤0.007), consistent with the prespecified ON/OFF sensitivity analysis.

**Conclusions:**

Under a harmonized OOF validation framework, the GA-derived composite risk score showed stable temporal discrimination and consistent overall predictive performance across three consecutive annual cohorts. These findings underscore the methodological importance of prespecified, standardized evaluation procedures and transparent uncertainty quantification when assessing reproducibility of risk stratification models in routine health screening data.

## Introduction

In preventive health screening, risk classification commonly relies on threshold-based evaluation of individual clinical indicators (eg, blood pressure, lipids, or HbA_1c_). While such approaches ensure procedural uniformity, they do not integrate multiple biological and lifestyle dimensions into a composite risk representation. Data-driven optimization approaches have therefore been proposed to enhance structural consistency and interpretability of health risk scoring.

Genetic algorithms (GAs), originally introduced by Holland [1] and further formalized by Goldberg [2], provide a flexible framework for feature weighting and optimization under cross-validated conditions. However, GA-based scoring models applied to real-world health checkup data require careful attention to reproducibility, methodological harmonization, and internal validation procedures. In particular, performance estimates may vary depending on whether preprocessing, optimization, and evaluation steps are strictly separated within a prespecified validation framework.

Bayesian estimation can be incorporated as an interpretive layer to express predicted risk probabilistically, aligning composite scores with calibrated predicted probabilities. Rather than emphasizing peak discrimination, evaluating temporal reproducibility under a standardized analytical framework is essential for ensuring methodological consistency.

The objective of this study was to evaluate the temporal reproducibility of a GA-derived composite health risk score across three consecutive annual cohorts (2021‐2023) using a prespecified, standardized out-of-fold validation pipeline.

## Methods

### Data Source and Participants

We analyzed a deidentified dataset from annual health checkups conducted at the Preventive Medicine Center (Ningen Dock Division), Yoshida Hospital, Keiyukai Medical Corporation (Asahikawa, Hokkaido, Japan). The analytic cohorts comprised examinees from 2021 (n=3744), 2022 (n=5153), and 2023 (n=5352), each analyzed as an independent annual cohort.

### Ethical Considerations

The study was approved by the Institutional Review Board of Yoshida Hospital, Keiyukai Medical Corporation (Approval No. 20251002001) and conducted in accordance with the Declaration of Helsinki. Data were deidentified prior to analysis. Written informed consent, including consent for secondary use of de-identified data, was obtained at the time of the health checkup. No additional interventions or participant contact occurred as part of this study.

### Measures and Preprocessing

Thirteen routine clinical indicators were included: BMI, waist circumference, systolic blood pressure, diastolic blood pressure, fasting plasma glucose, hemoglobin A1c (HbA_1c_), triglycerides, high-density lipoprotein cholesterol, low-density lipoprotein cholesterol, aspartate aminotransferase, alanine aminotransferase, γ-glutamyl transferase, and uric acid. Sex-specific thresholds were applied for waist circumference according to institutional criteria (see Table S2B in Multimedia Appendix 1).

Eight lifestyle questionnaire items were included (eg, smoking, alcohol consumption, breakfast habits, snacking, eating speed, mastication, physical activity/walking, and motivation for health improvement). Lifestyle questionnaire items were coded as binary variables according to the facility codebook. Variable definitions are provided in Table S2A in Multimedia Appendix 1.

Continuous variables were median-imputed and standardized within each training fold of each annual cohort. The imputation and standardization parameters were estimated within the training fold and applied to the corresponding held-out fold to prevent information leakage.

Indicator-level missingness rates are summarized in Table S1 in Multimedia Appendix 1. Missingness in indicators other than HbA_1c_ was low (<2% in each y).

### Outcome Definition

The primary outcome was defined as a composite abnormality label derived from routine health-check classification rules used in the screening program. For each clinical domain (eg, glucose metabolism, blood pressure, lipids, liver enzymes, and anthropometric indices), examinees were categorized according to prespecified threshold-based grades (A–D).

In the institutional screening system, grade A indicates no abnormality; grade B indicates mild abnormality typically requiring lifestyle guidance; grade C indicates follow-up or re-evaluation; and grade D indicates recommendation for further diagnostic evaluation or treatment.

The composite outcome was binarized using an OR rule: participants were labeled outcome-positive if any domain met or exceeded the predefined abnormality threshold (grade B or higher); otherwise, they were labeled outcome-negative. This definition was applied consistently across all annual cohorts to evaluate structural reproducibility of the operational framework rather than severity-specific prognostic discrimination. Detailed domain-specific thresholds corresponding to grade B or higher are provided in Table S2B in Multimedia Appendix 1.

The grading thresholds (A–D) were defined according to the standardized health-check classification framework established by the Japanese Society of Ningen Dock and Preventive Medicine, which is based on national health screening standards and specialty society guidelines. These classifications are widely used in routine health-check programs across Japan to guide follow-up recommendations (eg, observation, repeat testing, referral, or treatment). This study adopted these externally defined operational criteria without modification and dichotomized grade B or higher to capture any clinically relevant abnormality that warrants structured follow-up under this program. Participants categorized as grade E (under active treatment) were excluded from the outcome classification process.

### Handling of HbA1c Structural Missingness

HbA1c was structurally missing for a subset of participants due to the program’s screening policy-based test selection. Indicator-level missingness rates are summarized in Table S1 in Multimedia Appendix 1.

Because the composite outcome was defined using an OR rule across multiple domains, outcome ascertainment did not depend solely on HbA_1c_.

To evaluate robustness to structural HbA1c missingness, we conducted a prespecified sensitivity analysis excluding HbA1c from the predictor set while maintaining an identical evaluation pipeline (HbA_1c_ included vs excluded).

### Model Development and Evaluation

A composite score was generated from standardized features using a genetic algorithm (GA). All stochastic components were controlled by fixing the random seed (SEED=42) for both Python’s random module and NumPy. Fitness estimation used stratified K-fold cross-validation with shuffling (random_state=42).

The GA-derived composite score was subsequently entered into a logistic regression model to generate calibrated predicted probabilities within a prespecified out-of-fold (OOF) validation framework (Platt scaling [3]).

Bayesian updating was applied post hoc for interpretability purposes to the calibrated predicted probabilities and did not influence GA optimization or probability estimation.

For each fold, the model was trained on the training subset and evaluated on the corresponding held-out subset. OOF predictions were aggregated across folds to obtain a single internally validated prediction for each participant within each annual cohort.

### Discrimination and Calibration

Model discrimination was assessed using the area under the receiver operating characteristic curve (AUC) calculated from OOF predicted probabilities generated within the prespecified cross-validated pipeline.

Overall predictive performance was quantified using the OOF-based Brier score. Calibration was examined descriptively using calibration plots based on OOF predicted probabilities (Figure S1 in Multimedia Appendix 1). No additional recalibration or threshold optimization was performed beyond the prespecified validation framework.

### Bootstrap Uncertainty Estimation

To improve statistical transparency, 95% percentile bootstrap confidence intervals for OOF AUC and Brier score were computed using 2000 participant-level resamples within each annual cohort and HbA_1c_ condition (ON/OFF). Performance metrics were recalculated from the previously generated OOF predicted probabilities without refitting the model, thereby preserving internal validation and avoiding information leakage while maintaining the integrity of the original cross-validated predictions.

Year-stratified OOF performance estimates and confidence intervals are reported in Table S4 in Multimedia Appendix 1 and prespecified ON–OFF differences are summarized in Table S5 in Multimedia Appendix 1.

### Statistical Software

All analyses were performed in Python (version 3.13.5) using *scikit-learn* (version 1.6.1) [4] and DEAP (version 1.4) [5]. Analyses were conducted in a Jupyter-based environment (Anaconda distribution). Detailed GA configuration parameters, including population size, number of generations, weight initialization range, crossover and mutation settings, and random-seed control, are provided in Multimedia Appendix 1 (“Genetic Algorithm Implementation”) to facilitate reproducibility.

## Results

### Discrimination and Overall Predictive Performance Under Harmonized Out-of-Fold (OOF) Validation

Across the three annual cohorts, sample sizes ranged from 3744 to 5352 individuals, with outcome prevalence between 36.7% and 37.9%, indicating comparable class balance across years.

Under the standardized OOF validation framework, discrimination remained stable across cohorts. OOF AUC values were 0.810 (2021), 0.814 (2022), and 0.812 (2023), with overlapping 95 % bootstrap confidence intervals (Table 1). Brier scores ranged from 0.172 to 0.176 across cohorts, indicating stable overall predictive performance across years (Table 1).

**Table 1.** Discrimination and overall predictive performance under harmonized out-of-fold (OOF) validation (2021‐2023; primary model with HbA1c included). AUC and Brier score were calculated exclusively from OOF predicted probabilities generated within the prespecified cross-validation pipeline. Values in parentheses represent 95% percentile bootstrap confidence intervals based on 2000 resamples. Calibration plots based on OOF predicted probabilities are provided in Figure S1 in Multimedia Appendix 1. The prespecified HbA_1c_ ON/OFF sensitivity analysis and ON−OFF differences are summarized in Table S5 in Multimedia Appendix 1.

Year	Individuals, n	Outcome prevalence	OOF^a^ AUC^b^ (95% CI)	Brier score (95% CI)
2021	3744	0.375	0.810 (0.794‐0.820)	0.176 (0.170‐0.183)
2022	5153	0.379	0.814 (0.802‐0.825)	0.173 (0.168‐0.178)
2023	5352	0.367	0.812 (0.800‐0.824)	0.172 (0.166‐0.177)

a OOF: out-of-fold.

b AUC: area under the receiver operating characteristic curve.

Corresponding calibration plots are provided in Figure S1 in Multimedia Appendix 1.

In the prespecified HbA_1c_ ON/OFF sensitivity analysis, exclusion of HbA1c resulted in minimal changes in discrimination and Brier score (Table S5 in Multimedia Appendix 1), suggesting limited sensitivity of model performance to policy-driven structural HbA1c missingness under the harmonized OOF validation framework.

As the primary objective was to assess temporal reproducibility under standardized analytical procedures, formal hypothesis testing of between-year differences was not performed.

## Discussion

### Principal Findings

This study evaluated the temporal reproducibility of a genetic algorithm (GA)–derived composite health risk score across three consecutive annual health checkup cohorts under a prespecified OOF validation framework. Cross-validated OOF AUC values ranged from 0.810 to 0.814, with overlapping bootstrap confidence intervals, indicating stable discrimination under standardized analytical procedures. Earlier exploratory analyses yielded higher apparent AUC values; however, these were not derived exclusively from OOF predictions under the same pipeline and are therefore not presented as primary performance estimates. The primary contribution of this study is methodological: it demonstrates that a GA-derived score can achieve stable OOF discrimination across consecutive cohorts when preprocessing, optimization, and evaluation are uniformly applied, rather than emphasizing peak performance under heterogeneous analytical conditions.

Because performance estimates were based exclusively on OOF predicted probabilities, the evaluation preserved internal validation and minimized information leakage. Variability observed in earlier exploratory analyses likely reflected differences in analytical procedures rather than underlying cohort characteristics, highlighting the importance of consistent preprocessing, optimization, and evaluation when assessing artificial intelligence–based risk stratification models.

Importantly, the outcome definition reflects operational screening classification rather than confirmed clinical diagnoses. The composite label was constructed using threshold-based OR combinations across correlated clinical domains, and the dominant abnormality domain contributing to classification may vary across cohorts depending on distributional shifts. In this context, AUC values represent the model’s ability to consistently reconstruct the structured screening framework under harmonized analytical conditions rather than discrimination of severity-specific disease states.

Methodologically, GA optimization produced a composite score from standardized predictors, which was subsequently mapped to calibrated predicted probabilities using logistic regression. Bayesian updating was applied post hoc as an interpretability add-on to the calibrated predicted probabilities and did not influence GA optimization or probability estimation. The explicit specification of evolutionary hyperparameters, weight initialization range, and random-seed control further strengthens the reproducibility of the optimization procedure and reduces the likelihood that the observed discrimination reflects stochastic artifacts.

The prespecified HbA1c ON/OFF sensitivity analysis showed minimal changes in AUC and Brier score, suggesting limited sensitivity to policy-driven structural HbA1c missingness under the standardized evaluation framework; however, re-optimizing GA weights under an altered feature set represents a distinct modeling exercise.

This study has limitations. It was conducted at a single center in Japan using an occupational health checkup population. The outcome was cross-sectional, and prospective or external validation was not performed. In addition, because grade B or higher includes heterogeneous categories with varying clinical implications (ranging from lifestyle guidance to referral for diagnostic evaluation), model discrimination reflects detection of operational abnormality rather than exclusively high-severity disease states. Furthermore, because the outcome was defined using threshold-based criteria that partially overlap with included predictors, discrimination should be interpreted as reconstruction of the operational classification within the same workflow rather than independent prognostic discrimination. This endpoint is an operational classification designed for workflow consistency, not a clinically adjudicated diagnosis. Accordingly, the present OOF-based results provide an internally validated assessment of temporal reproducibility within this screening system.

### Conclusions

In conclusion, under a prespecified, harmonized OOF validation framework within a single institutional screening system, the GA-derived composite risk score demonstrated stable temporal discrimination and consistent overall predictive performance across three consecutive annual cohorts. These findings support methodological reproducibility and structural consistency under standardized analytical procedures. However, the present results do not establish external generalizability or clinical effectiveness, and independent external and prospective validation is required before broader clinical implementation can be inferred.

## Supplementary material

10.2196/85659 Multimedia Appendix 1 Supplementary materials including Tables S1–S5, additional methodological details (GA implementation and Bayesian risk update), calibration metrics, and prespecified HbA_1c_ ON/OFF sensitivity analyses.

## Abbreviations

AUC: area under the receiver operating characteristic curve
GA: genetic algorithm
HbA_1c_: hemoglobin A1c
OOF: out-of-fold

## References

[1] Holland JH (1975). Adaptation in Natural and Artificial Systems.

[2] Goldberg DE (1989). Genetic Algorithms in Search, Optimization, and Machine Learning.

[3] Platt J (1999). Advances in Large Margin Classifiers.

[4] Pedregosa F (2011). Scikit-learn: machine learning in Python. J Mach Learn Res.

[5] Fortin FA (2012). DEAP: evolutionary algorithms made easy. J Mach Learn Res.

