# Supplementary material for: Temporal Reproducibility of a Genetic Algorithm–Derived Health Risk Score: Standardized Out-of-Fold Validation Framework (2021-2023)
Source: JMIR Bioinform Biotechnol. 2026 Apr 21;7:e85659. doi: 10.2196/85659 (PMC13099121; doi:10.2196/85659)

**Supplementary Materials**

**Supplementary Table S1.** Indicator-level missingness rates by year (2021–2023).

Indicator-level missingness reflects the proportion (%) of participants with missing values for each biomarker within each annual cohort. HbA1c missingness represents policy-driven structural missingness related to the screening program design.

| Indicator | 2021 (%) | 2022 (%) | 2023 (%) |
| --- | --- | --- | --- |
| Body mass index (BMI) | 0.5 | 0.4 | 0.4 |
| Waist circumference | 1.3 | 0.9 | 0.7 |
| Systolic blood pressure (SBP) | 0.5 | 0.4 | 0.4 |
| Diastolic blood pressure (DBP) | 0.5 | 0.4 | 0.4 |
| Fasting plasma glucose (FPG) | 1.3 | 0.9 | 0.7 |
| Hemoglobin A1c (HbA1c) | 31.9 | 30.8 | 31.3 |
| Triglycerides (TG) | 1.3 | 0.9 | 0.7 |
| HDL cholesterol | 1.3 | 0.9 | 0.7 |
| LDL cholesterol | 1.3 | 0.9 | 0.7 |
| Aspartate aminotransferase (AST) | 1.3 | 0.9 | 0.7 |
| Alanine aminotransferase (ALT) | 1.3 | 0.9 | 0.7 |
| Gamma-glutamyl transferase (GGT, γ-GTP) | 1.3 | 0.9 | 0.7 |
| Uric acid (UA) | 1.3 | 0.9 | 0.7 |

All non-HbA1c indicators had less than 2% missingness across all cohorts.

**Supplementary Table S2A.** Definitions of measures used in model development (clinical indicators and lifestyle factors).

This table summarizes variable names, measurement definitions, units, and coding schemes used in the genetic algorithm (GA) modeling pipeline. Clinical indicators were treated as continuous variables and were median-imputed and standardized within each training fold prior to GA optimization. Lifestyle factors were derived from the facility’s standardized health checkup questionnaire and coded as binary variables (1 = yes, 0 = no) according to the facility’s official codebook.

**Clinical Indicators**

| Variable | Definition / Measurement | Unit |
| --- | --- | --- |
| Body mass index (BMI) | Weight (kg) / height (m²) | kg/m² |
| Waist circumference | Measured at the level of the umbilicus | cm |
| Systolic blood pressure (SBP) | Mean of two seated measurements | mmHg |
| Diastolic blood pressure (DBP) | Mean of two seated measurements | mmHg |
| Fasting plasma glucose (FPG) | Enzymatic method (fasting state) | mg/dL |
| Hemoglobin A1c (HbA1c) | NGSP standard | % |
| Triglycerides (TG) | Enzymatic method | mg/dL |
| HDL cholesterol | Enzymatic method | mg/dL |
| LDL cholesterol | Enzymatic method | mg/dL |
| Aspartate aminotransferase (AST) | Enzymatic method | U/L |
| Alanine aminotransferase (ALT) | Enzymatic method | U/L |
| Gamma-glutamyl transferase (GGT, γ-GTP) | Enzymatic method | U/L |
| Uric acid (UA) | Enzymatic colorimetric method | mg/dL |

**Lifestyle Factors**

| Variable | Definition | Coding |
| --- | --- | --- |
| Smoking | Current smoker | 1 = yes, 0 = no |
| Alcohol consumption | ≥2 units/day or ≥3 times/week (1 unit ≈ 20 g ethanol; per facility codebook; aligned with Japanese health checkup guidance) | 1 = yes, 0 = no |
| Breakfast habits | Regular breakfast (≥5 days/week) | 1 = yes, 0 = no |
| Snacking | Frequent snacking between meals | 1 = yes, 0 = no |
| Eating speed | Self-rated fast eating | 1 = yes, 0 = no |
| Mastication | Reports sufficient chewing | 1 = yes, 0 = no |
| Physical activity or walking | ≥30 min/day regular activity | 1 = yes, 0 = no |
| Motivation for health improvement | Self-reported intention to maintain/improve health | 1 = yes, 0 = no |

Note: The composite outcome was defined separately using the institutional screening classification system (grades A–D) and an OR rule across domains. Predefined clinical cutoffs were not used in GA optimization. Predictors were entered exclusively as continuous (clinical indicators) or binary (lifestyle factors) inputs in GA optimization and were not thresholded or dichotomized before model training.

**Supplementary Table S2B.** Institutional screening thresholds used for rule-based composite outcome definition (grade B or higher).

| Domain | Indicator | Threshold for grade ≥B |
| --- | --- | --- |
| Glucose metabolism | FPG | ≥100 mg/dL |
| Glucose metabolism | HbA1c | ≥5.6% |
| Blood pressure | SBP | ≥130 mmHg |
| Blood pressure | DBP | ≥85 mmHg |
| Lipids | LDL cholesterol | ≥120 mg/dL |
| Lipids | HDL cholesterol | ≤39 mg/dL |
| Lipids | Triglycerides | ≥150 mg/dL |
| Anthropometry | BMI | ≤18.4 kg/m² or ≥25 kg/m² |
| Anthropometry | Waist circumference (male) | ≥85 cm |
| Anthropometry | Waist circumference (female) | ≥90 cm |
| Liver enzymes | AST | ≥31 U/L |
| Liver enzymes | ALT | ≥31 U/L |
| Liver enzymes | GGT | ≥51 U/L |
| Uric acid | UA | ≥7.1 mg/dL |

Thresholds are defined according to the standardized grading framework of the Japanese Society of Ningen Dock and Preventive Medicine and were used exclusively for outcome labeling. The composite outcome was defined as grade B or higher in any domain (OR rule). For glucose metabolism, grade ≥B corresponds to FPG ≥100 mg/dL and/or HbA1c ≥5.6%, consistent with the institutional classification framework in effect during the study period.

**Supplementary Methods**

**Bayesian Risk Update (Interpretability Add-On)**

To contextualize model discrimination within a screening workflow, we applied a post hoc Bayesian risk update. For illustration, a fixed prior probability (p₀ = 0.33) was used in Supplementary Table S3. This calculation was performed for interpretability only and did not influence GA optimization or probability estimation.

At a prespecified operating point (Youden’s J), sensitivity (Se) and specificity (Sp) were obtained from out-of-fold (OOF) predictions and used to compute likelihood ratios:

LR⁺ = Se / (1 − Sp)

LR⁻ = (1 − Se) / Sp

Pre-test odds were defined as:

odds₀ = p₀ / (1 − p₀)

Post-test odds were computed as:

For a positive screen: odds⁺ = odds₀ × LR⁺

For a negative screen: odds⁻ = odds₀ × LR⁻

Post-test probability was then derived as:

p = odds / (1 + odds)

This Bayesian update is conceptually distinct from probability calibration (Platt scaling [4]). Calibration maps model outputs to probabilities aligned with observed event rates, whereas the Bayesian update translates an operating threshold (via Se/Sp and likelihood ratios) into an interpretable absolute post-test risk for decision support in screening workflows.

**Supplementary Table S3.**  Bayesian update examples at the prespecified Youden threshold (illustrative prior).

Sensitivity and specificity were calculated from out-of-fold (OOF) predictions at the prespecified Youden threshold. The prior probability (p₀) is illustrative and does not affect model calibration or discrimination estimates.

| Year | Prior Probability (p₀) | Sensitivity (Se) | Specificity (Sp) | LR⁺ | LR⁻ | Post-test Probability (Positive) |
| --- | --- | --- | --- | --- | --- | --- |
| 2021 | 0.33 | 0.84 | 0.85 | 5.60 | 0.19 | 0.73 |
| 2022 | 0.33 | 0.83 | 0.84 | 5.19 | 0.20 | 0.72 |
| 2023 | 0.33 | 0.82 | 0.83 | 4.82 | 0.22 | 0.70 |

**Genetic Algorithm Implementation (DEAP Library)**

Features were median-imputed and standardized within each training fold prior to optimization. GA optimization was implemented in Python (Anaconda/Jupyter) using scikit-learn [3] and DEAP [5].

Key hyperparameters:

- Population size: 50
- Maximum generations: 100
- Crossover probability: 0.7
- Mutation probability: 0.2
- Per-gene independent mutation probability (indpb): 0.2

Feature weights were initialized from a uniform distribution within the range [0.0, 3.0]. Gaussian mutation (μ=0, σ=0.5, indpb=0.2) was applied without explicit bounding constraints during evolution. The indpb parameter specifies the probability that each individual feature weight is independently mutated during a mutation event.

Random seed control. All stochastic components were controlled by initializing Python’s random module and NumPy with SEED=42 prior to GA execution. Stratified K-fold cross-validation also used random_state=42, ensuring full reproducibility of evolutionary optimization and fitness estimation.

**Supplementary Table S4.** OOF discrimination and overall performance by year and HbA1c condition (ON/OFF).

Values are median (95% percentile bootstrap CI) based on 2,000 participant-level resamples of out-of-fold (OOF) predicted probabilities. Positive outcome rate (pos_rate) denotes the proportion of outcome-positive participants within each annual cohort.

| Year | Mode | n | pos_rate | AUC (median [95% CI]) | Brier (median [95% CI]) |
| --- | --- | --- | --- | --- | --- |
| 2021 | OFF | 3744 | 0.375 | 0.807 (0.794–0.820) | 0.178 (0.170–0.185) |
| 2021 | ON | 3744 | 0.375 | 0.810 (0.794–0.820) | 0.176 (0.170–0.183) |
| 2022 | OFF | 5153 | 0.379 | 0.809 (0.798–0.820) | 0.176 (0.169–0.182) |
| 2022 | ON | 5153 | 0.379 | 0.814 (0.802–0.825) | 0.173 (0.168–0.178) |
| 2023 | OFF | 5352 | 0.367 | 0.806 (0.795–0.818) | 0.175 (0.168–0.181) |
| 2023 | ON | 5352 | 0.367 | 0.812 (0.800–0.824) | 0.172 (0.166–0.177) |

**Supplementary Table S5.** Sensitivity analysis of HbA1c inclusion (ON − OFF) based on OOF bootstrap.

Δ denotes ON − OFF (HbA1c included minus excluded). Values are median differences (95% percentile bootstrap CI) based on 2,000 resamples of OOF predicted probabilities. Positive ΔAUC and negative ΔBrier indicate improved discrimination and overall predictive performance (Brier score) when HbA1c is included.

| Year | N | ΔAUC (ON − OFF), median (95% CI) | ΔBrier (ON − OFF), median (95% CI) |
| --- | --- | --- | --- |
| 2021 | 3744 | 0.005 (0.002–0.008) | -0.002 (-0.004–-0.001) |
| 2022 | 5153 | 0.007 (0.004–0.010) | -0.003 (-0.004–-0.002) |
| 2023 | 5352 | 0.007 (0.005–0.010) | -0.004 (-0.005–-0.003) |

**Supplementary Figure S1.** Calibration of OOF predicted probabilities (HbA1c ON vs OFF; pooled 2021–2023 shown separately).

Calibration plots based on out-of-fold (OOF) predicted probabilities for HbA1c-included (ON) and HbA1c-excluded (OFF) pipelines. Each colored line represents one annual cohort (2021–2023). Predicted probabilities were grouped into deciles (quantile bins) and plotted against observed outcome proportions. Because predictions are OOF, each participant’s probability was generated from models trained without that participant, supporting internal validation without information leakage. The blue dashed diagonal line indicates perfect calibration. ON and OFF curves show similar calibration patterns, consistent with minimal changes in discrimination and overall predictive performance under HbA1c exclusion.


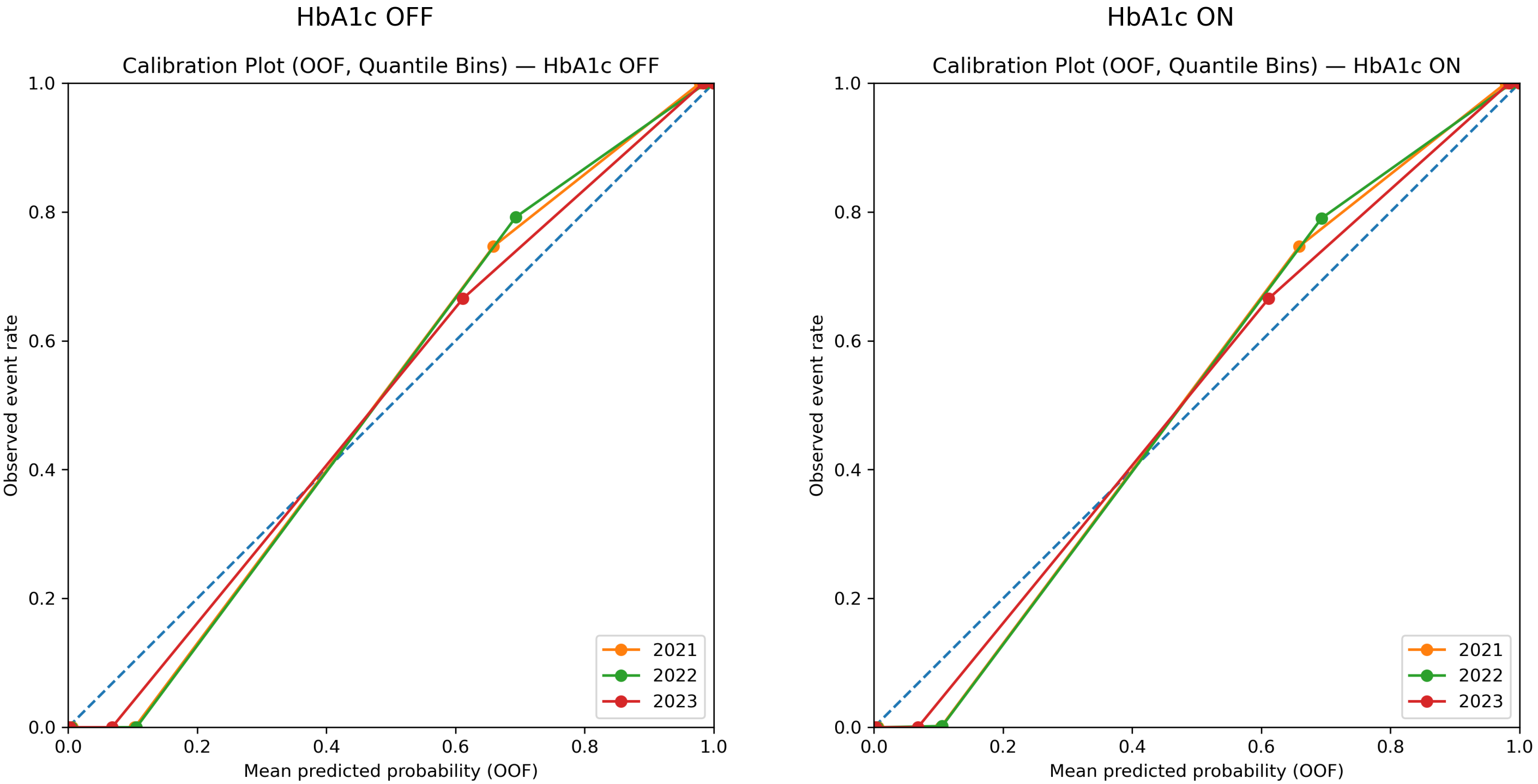

Supplement: Multimedia Appendix 1 [file bioinform-v7-e85659-s001.docx]
